# First Molecular Characterization of Bovine Leukemia Virus Infections in the Caribbean

**DOI:** 10.1371/journal.pone.0168379

**Published:** 2016-12-15

**Authors:** Yi Yang, Patrick John Kelly, Jianfa Bai, Rong Zhang, Chengming Wang

**Affiliations:** 1 Jiangsu Co-Innovation Center for the Prevention and Control of Important Animal Infectious Diseases and Zoonoses, Yangzhou University College of Veterinary Medicine, Yangzhou, Jiangsu, China; 2 Department of Veterinary Diagnostic Laboratory, College of Veterinary Medicine, Kansas State University, Kansas, Kansas, United States of America; 3 Ross University School of Veterinary Medicine, Basseterre, Saint Kitts and Nevis; 4 Department of Pathobiology, College of Veterinary Medicine, Auburn University, Auburn, Alabama, United States of America; University of Alabama at Birmingham, UNITED STATES

## Abstract

Bovine leukemia virus (BLV) is a retrovirus that causes enzootic bovine leucosis. To investigate the presence and genetic variability of BLV in the Caribbean for the first time, we preformed fluorescence resonance energy transfer (FRET)-PCR for the *pol* of BLV on DNA from whole blood of cattle from Dominica, Montserrat, Nevis and St. Kitts. Standard PCRs with primers for the *env* were used for phylogenetic analysis of BLV in positive animals. We found FRET-PCR positive cattle (12.6%, 41/325) on Dominica (5.2%; 4/77) and St. Kitts (19.2%; 37/193) but not on Montserrat (0%, 0/12) or Nevis (0%, 0/43). Positive animals were cows on farms where animals were raised intensively. Phylogenetic analysis using the neighbor-joining (NJ) method on partial and full-length *env* sequences obtained for strains from Dominica (n = 2) and St. Kitts (n = 5) and those available in GenBank (n = 90) (genotypes 1–10) revealed the Caribbean strains belonged to genotype 1 (98–100% sequence homology). Ours is the first molecular characterization of BLV infections in the Caribbean and the first description of genotype 1 in the region.

## Introduction

The bovine leukemia virus (BLV) is a member of the Retroviridae and the agent of enzootic bovine leucosis (EBL). While most infected cattle become asymptomatic carriers, approximately 30% develop persistent lymphocytosis and 5% die from malignant lymphoma [1]. Further, BLV infection is associated with decreased milk yields, changes in milk composition and shortened life-span [1–4].

As with other retroviruses, the BLV genome contains *gag*, *pol* and *env* structural genes and *Tax*, *Rex*, *R3* and *G4* regulatory genes. The *env* encodes a mature surface glycoprotein (gp51) and a transmembrane protein (gp30) [5] which is involved in viral infectivity [6–8]. Based on *env* sequences, various genetic groupings have been described [9–16]. In 2009, Rodriguez et al. [17] used new and existing sequence data to differentiate BLV into seven genotypes and subsequently three new genotypes were described in Croatia [18], Bolivia [19], Eastern Europe and Siberia [20], Thailand [21] and Myanmar [22] to bring the total number of described genotypes to 10. Although BLV has been described on all continents, there is little information on BLV infections in the Caribbean region. Although there is anecdotal evidence that BLV is present on a number of Caribbean islands (Invasive Species Compendium, bovine enzootic leukemia. Retrieved 2016, October 14 from www.cabi.org/isc/datasheet/91731#20056913112), there have been no molecular studies to definitively diagnose infections and characterize the genotypes that might be present. To provide such data, we tested cattle from four Caribbean islands for BLV provirus using fluorescence resonance energy transfer (FRET)-PCR. Phylogenetic analysis was performed on BLV-positive samples.

## Materials and Methods

### Blood samples

**Fig 1:**
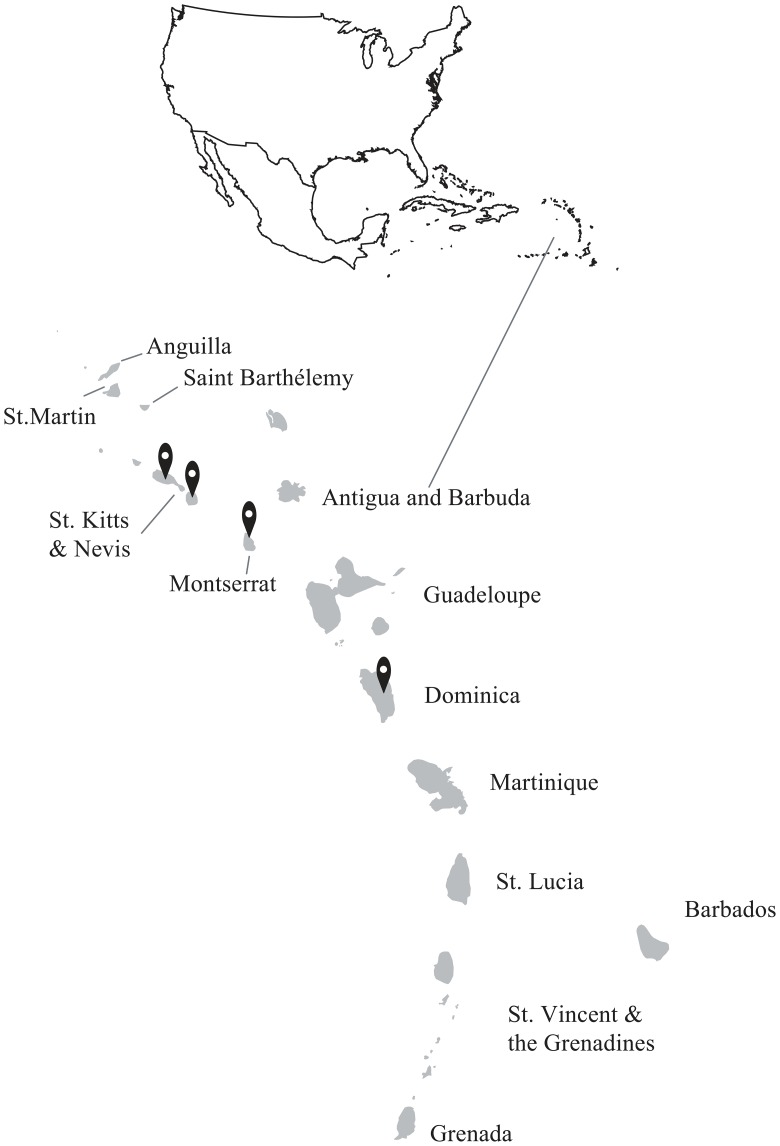
The islands of Dominica, Montserrat, Nevis and St. Kitts from which cattle were sampled for the study. A total of 325 blood samples were collected from Dominica (N = 77; 15.42°N, 61.33°W), Montserrat N = 12;16.75°N, 62.20°W), Nevis (N = 43; 17.15°N, 62.58°W) and St. Kitts (N = 193; 17.25°N, 62.67°W).

Convenience whole blood samples were collected into EDTA from apparently healthy, locally bred, adult cattle on the Caribbean islands of Dominica, Montserrat, Nevis and St. Kitts (Fig 1) as described previously [23–24]. The study was reviewed and approved by the Institutional Animal Care and Use Committee of Ross University School of Veterinary Medicine, St. Kitts. Owners of the animals provided consent for blood samples to be collected.

### DNA extraction

Buffy coats were stored at -20°C until thawed at room temperature and genomic DNA extracted from 200 μL aliquots using the QIAamp DNA Blood Mini Kit (QIAGEN, Valencia, CA, USA) according to the manufacturer’s instructions. The DNA was eluted in 200 μL washing buffer and shipped to Yangzhou University College of Veterinary Medicine of Jiangsu province, China at room temperature where it was frozen at -80°C until PCRs were performed.

### FRET-PCR to detect BLV

The BLV FRET-PCR was performed as described previously [3] in a LightCycler 480 II real-time PCR platform to detect BLV. In brief, the *gag* gene sequences of representative BLV isolates were obtained from GenBank and the Clustal multiple alignment algorithm was used to identify a highly conserved region of the proviral polymerase DNA gene common to all the above BLV gene sequences. The BLV FRET-qPCR established amplifies a 187-bp target. Thermal cycling consisted of a 2-min denaturation step at 95°C followed by 18 high-stringency step-down thermal cycles. A plasmid containing the anticipated BLV *pol* FRET-PCR amplicon region synthesized by Genscript (Nanjing, Jiangsu, China) and dissolved in T_10_E_0.1_ buffer was used as the positive control.

### BLV *env* gene amplification and sequencing

PCR amplification of the full-length BLV *env* (1,548 bp) was performed with two pairs of primers we designed for the study: primer pair one—forward primer 5’-TGTGGGTTCCCTGGCGTTT-3’ and reverse primer 5’-GAGGTGAGTCTCTGATGGCTAAGG-3’; primer pair 2—forward primer 5’-GTCAGTGGGGCTCACTGGAATT-3’ and reverse primer 5’-GGCGTAAAAAGCGGAAGCTG-3’. Amplicons were gel purified with the QIAquick Gel Extraction Kit as described before [25] and sequenced at the Genomic Sequencing Laboratory (GBI, Shanghai, China). Sequence data were assembled and analyzed with Vector NTI Advance 11 software (Thermo Fisher Scientific).

### Phylogenetic analysis of BLVs

The BLV *env* sequences we obtained were aligned using CLUSTAL W in MEGA 7 [26] along with those of BLV strains found on GenBank from around the world. Neighbor-joining (NJ) phylogenetic trees were constructed using the Tamura-Nei model [20, 27] and robustness of clusters assessed by bootstrapping 1,000 replicates. Maximum-likelihood (ML) phylogenetic analysis was preformed to confirm the results.

### Nucleotide and amino acid distance of BLVs

The nucleotide and deduced amino acid distance of BLVs within (intra-genotype) and among (inter-genotype) genotypes were estimated using the Tamura-Nei model and Poisson model in MEGA 7 by bootstrapping 1,000 replicates

## Results

### Prevalence of BLV in Caribbean islands

We examined a total of 325 samples of which 41 (12.6%) were positive in our BLV *pol* FRET-PCR. Positive animals were identified on Dominica (5.2%, 4/77) and St. Kitts (19.2%, 37/193) but not on Montserrat (0%, 0/12) or Nevis (0%, 0/43). The positive animals from Dominica and St. Kitts were cows from relatively large herds of beef cattle reared intensively; Government Central Livestock Farm, Dominica and Ross University School of Veterinary Medicine beef herd, St. Kitts. Copy numbers in positive animals ranged from 20 copies/ ml to 1,045,562 copies/ ml (mean 137,165 copies/ ml and median 8,427 copies/ ml) (Table 1).

**Table 1:** Molecular detection of BLV Provirus DNA in Dominica, St. Kitts, Montserrat and Nevis.

	% positive, N	Copy number range (per ml)	Copy number mean (per ml)	Copy number median (per ml)
**Dominica**	5.2%, 4/77	20–1,045,562	379,628	236,464
**St. Kitts**	19.2%, 37/193	20–649,480	110,953	8,427
**Montserrat**	0%, 0/12	N/A	N/A	N/A
**Nevis**	0%, 0/43	N/A	N/A	N/A
**Total**	12.6%, 41/325	20–1,045,562	137,165	8,427

### Phylogenetic analysis of partial and full-length BLV *env* sequences

**Fig 2:**
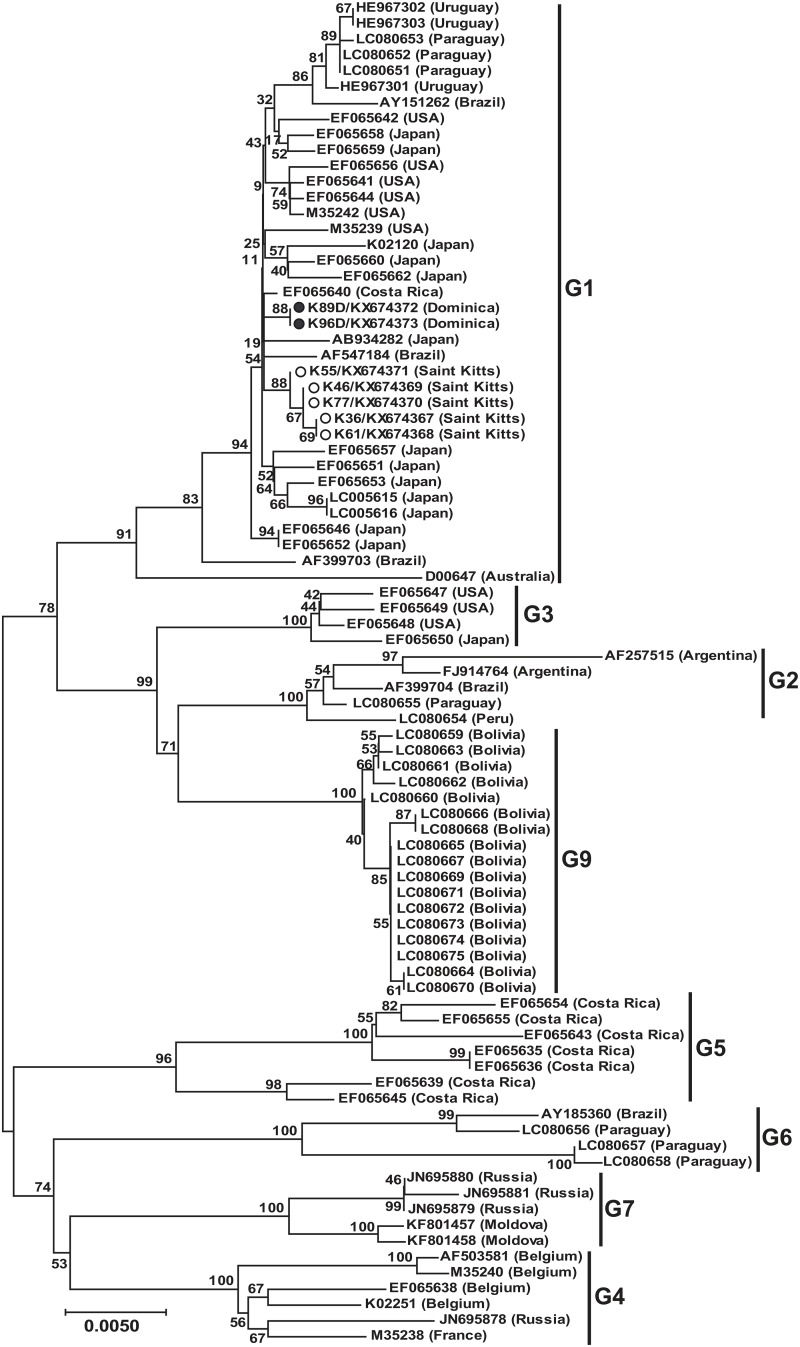
Neighbor-joining phylogenetic tree based on sequences of the BLV *env* (1,548 bp) from the Caribbean and other areas around the world. Strains identified in our study from Dominica are identified with filled (●) circles and those from St. Kitts by open (○) circles. Genotypes shown on the right are according to Lee et al., Polat et al., and Moratorio et al. [21, 27, 28]. Numbers at the branches show bootstrap support (1,000 replicates). The bar at the bottom of the figure denotes distance.

**Fig 3:**
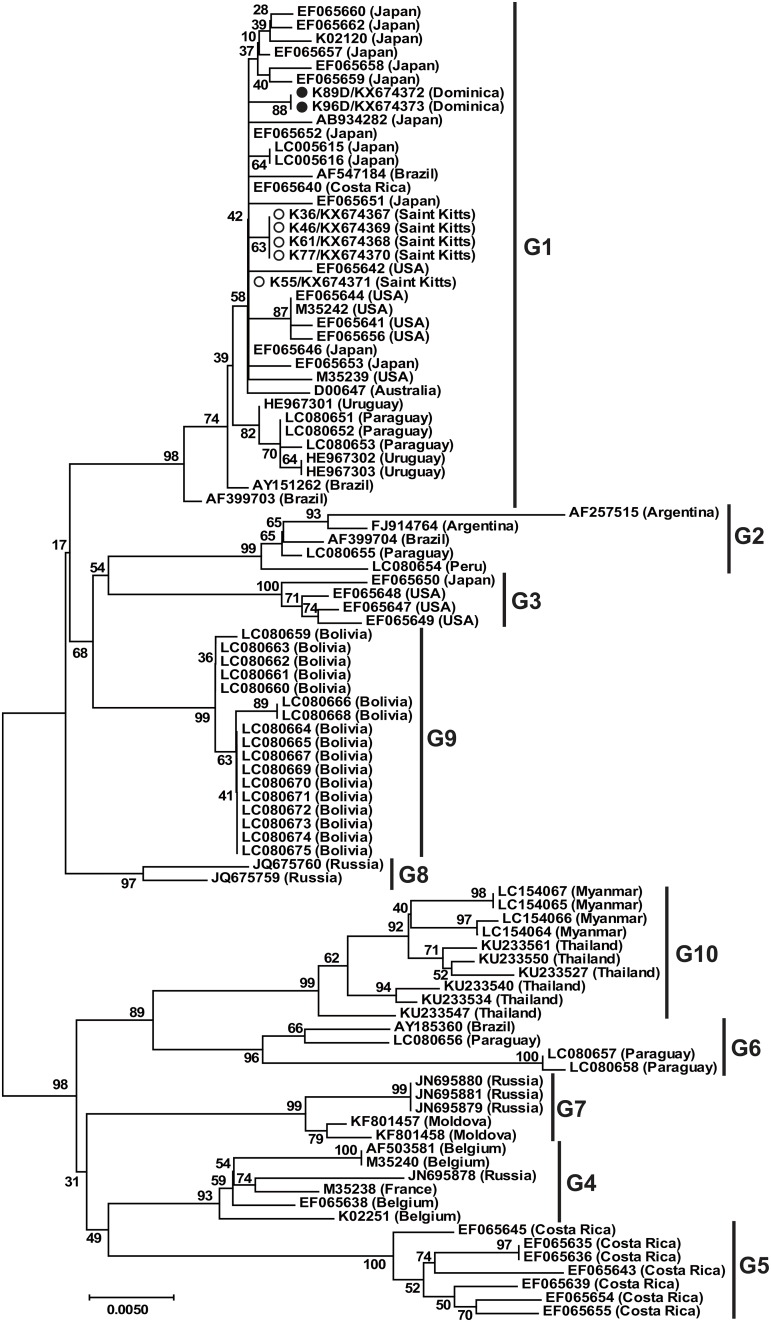
Neighbor-joining phylogenetic tree based on sequences of the BLV *env-gp51* (807 bp) from the Caribbean and other areas around the world. Strains identified in our study from Dominica are identified with filled (●) circles and those from St. Kitts by open (○) circles. Genotypes shown on the right are according to Lee et al., Polat et al., and Moratorio et al. [21, 22, 27, 28]. Numbers at the branches show bootstrap support (1,000 replicates). The bar at the bottom of the figure denotes distance.

We obtained full-length *env* sequences for two of the BLV *pol* FRET-PCR positive animals from Dominica and five from St. Kitts. Generally, the copy numbers of the samples from which full-length sequences were derived were high, 157 copies/ ml to 472,835 copies/ ml, mean 293,010 copies/ ml, median 269,519 copies/ ml. A NJ phylogenetic tree based on full-length (1,548 bp) *env* sequences of our Caribbean strains and 78 reference strains representing genotypes 1–7, 9 from 14 countries that were available from GenBank, showed all the Caribbean strains belonged to genotype 1 (Fig 2). To confirm our result, we also constructed a NJ phylogenetic tree based on a partial BLV *env* sequences (807 bp, including the gp51 and gp30g encoding regions) of the Caribbean strains and also those of 90 reference strains from GenBank which represent all 10 BLV genotypes [28] (Fig 3). This second analysis also showed that the Caribbean strains clustered within genotype 1.

The mean distance of the nucleotides and deduced amino acids were 0.003±0.002 and 0.002±0.001, respectively, for the seven Caribbean BLV strains identified in this study (Table 2). The Caribbean stains had between 95.3% (LC080658) and 99.8% (EF065640) similarity with the BLV strains from eight genotypes found in GenBank (S1 Table). However, all had greatest similarity with GenBank references strains belonging to genotype 1, from 97.7% (D00647) to 99.8% (EF065640). Likewise, the similarities of the deduced amino acid sequences of the Caribbean strains and the GenBank reference strains varied from 95.3% (LC080658) to 99.8% (EF065640) with the greatest similarities being with genotype 1 strains, mainly from 98.0% (D00647) to 100% (EF065040, EF065641, EF065042, EF065651, EF065657 and EF065660) (S1 Table).

**Table 2:** Mean nucleotide and amino acid distances in full-length *env* sequences of Caribbean strains and strains from GenBank representing eight BLV genotypes.

Strain	KX674367-KX674373	G1	G2	G3	G4	G5	G6	G7	G9
**KX674367-KX674373**	**0.003±0.002**^c^ **0.002±0.001**^d^	0.006±0.002^b^	0.030±0.006	0.022±0.006	0.027±0.006	0.034±0.007	0.032±0.007	0.026±0.006	0.022±0.006
**G1**	0.006±0.001^a^	**0.007±0.001** **0.007±0.001**	0.030±0.006	0.023±0.006	0.027±0.006	0.034±0.007	0.032±0.007	0.026±0.006	0.022±0.006
**G2**	0.031±0.004	0.032±0.004	**0.019±0.004** **0.010±0.002**	0.017±0.004	0.032±0.006	0.042±0.007	0.038±0.007	0.033±0.007	0.018±0.004
**G3**	0.028±0.004	0.029±0.004	0.025±0.004	**0.001±0.001** **0.005±0.001**	0.024±0.006	0.035±0.007	0.031±0.007	0.026±0.007	0.012±0.004
**G4**	0.035±0.004	0.036±0.004	0.038±0.004	0.033±0.004	**0.011±0.003** **0.015±0.002**	0.032±0.006	0.027±0.006	0.023±0.006	0.025±0.006
**G5**	0.038±0.004	0.039±0.004	0.044±0.005	0.042±0.005	0.038±0.004	**0.016±0.004** **0.016±0.002**	0.037±0.007	0.032±0.006	0.037±0.007
**G6**	0.043±0.005	0.044±0.005	0.048±0.005	0.042±0.005	0.041±0.004	0.047±0.004	**0.013±0.004** **0.018±0.003**	0.024±0.006	0.030±0.007
**G7**	0.035±0.005	0.036±0.005	0.041±0.005	0.038±0.005	0.034±0.005	0.043±0.005	0.044±0.005	**0.008±0.003** **0.008±0.002**	0.027±0.007
**G9**	0.028±0.004	0.030±0.004	0.023±0.003	0.022±0.003	0.035±0.005	0.043±0.005	0.043±0.005	0.040±0.005	**0.001±0.001** **0.002±0.001**

^a^ Left lower diagonal: nucleotide distance among (inter-genotype) BLV genotypes and our isolates.

^b^ Right upper diagonal: amino acid distance among (inter-genotype) BLV genotypes and our isolates.

^cd^ The values in bold along the diagonal are the distance (intra-genotype) of the nucleotides (above) and amino acids (below) between the Caribbean strains and those in GenBank

### Alignment of *env* nucleotide and deduced amino acid sequences of Caribbean BLV strains and reference strains in GenBank

**Fig 4:**
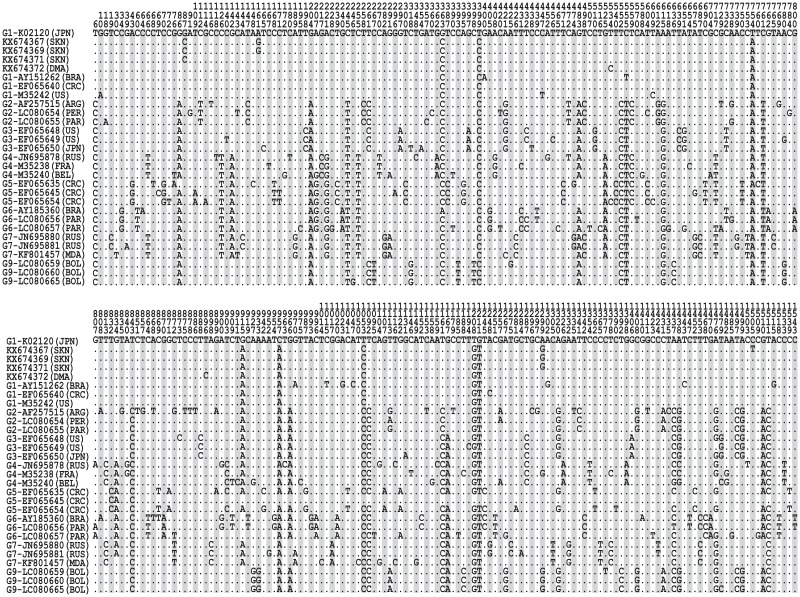
Alignment of BLV *env* nucleotide between 25 reference strains from GenBank and four Caribbean strains (KX674367, KX674369, KX674371 and KX674373). Numbers above the sequences are nucleotide number and amino acid residue number indicated by the *env* gene of K02120. The countries of the strains are marked with abbreviations in parentheses to the right of the GenBank accession numbers. Dots indicate nucleotides or amino acids identical to the reference sequence.

**Fig 5:**
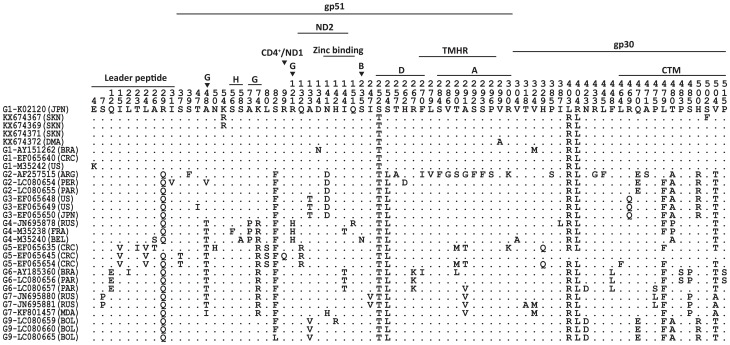
Alignment of BLV *env* amino acid sequences between 25 reference strains from GenBank and four Caribbean strains (KX674367, KX674369, KX674371 and KX674373). Numbers above the sequences are nucleotide number and amino acid residue number indicated by the *env* gene of K02120. The countries of the strains are marked with abbreviations in parentheses to the right of the GenBank accession numbers. Dots indicate nucleotides or amino acids identical to the reference sequence. Labeled lines and ▼ indicate the position of identified glycoproteins, epitopes, the transmembrane hydrophobic region (TMHR) and the cytoplasmic domain of the transmembrane protein (CTM).

The sequences obtained in our study were submitted to GenBank (accession numbers KX674367-KX674373). When we aligned the nucleotide (Fig 4) and deduced amino acid (Fig 5) sequences with those of 24 reference strains representing eight BLV genotypes from around world, we found the strains from St. Kitts had unique silent substitutions in the third base of residue 29 (nucleotide 87) and a strain from Dominica (KX674372) had a unique silent substitution in the third base of residue 178 (nucleotide 534). Two strains from St. Kitts (KX674367 and KX674369) had a conservative substitution [29] in the second base of residue 54 (nucleotide 161) and one from Dominica (KX674372) had a conservative substitution [29] in the second base of residue 299 (nucleotide 896). Interestingly, one strain from St. Kitts (KX674367) had a non-conservative [29] substitution in the second base of 502 (nucleotide 1,505).

## Discussion

Our study shows for the first time that cattle in the Caribbean are infected with BLV. Although we used only convenience samples, we found cattle from two of the four islands we studied were infected indicating infections might be prevalent in the region. While it is possible that the negative status of the two islands resulted from the small sample size we studied, it appears unlikely that it was due to lack of sensitivity in the tests we used. The FRET-PCR we used for screening has been shown to detect very low copy numbers [30] and we were able to detect positive samples with as low as 20 copies /ml in the study.

It is noteworthy that on the islands where we established BLV was present, the only animals we found positive were cows that belonged to relatively large beef herds that were raised intensively. We did not find any positive animals belonging to small holders, arbitrarily defined as owners with fewer than 12 cattle which are raised extensively under typical Caribbean management conditions [23]. While perinatal transmission of BLV is important [31], the virus can also be spread in leukocytes in blood, secretions and excretions [32]. The close contact required for such spread is less likely in cattle kept in the Caribbean as they are mostly allowed to wander freely. Further, there are few management procedures performed on Caribbean cattle, such as rectal palpation, dehorning, ear-tagging, tattooing, and vaccinations, which also promote the spread of infections [33–34]. Further studies are needed to more accurately determine the prevalence of infections on different islands in the Caribbean and also to determine the risk factors for infection that might be specific for the management systems employed for cattle in the region. Such data will be important in establishing policies for controlling infections in the region. As there is no vaccine against BLV, control might have to be by eliminating carrier animals which has proved successful in Western Europe [35].

Phylogenetic analysis of the full-length *env* sequences we obtained and our amino acid data showed clearly that the BLV we detected belonged to genotype 1. This genotype, along with genotype 3, is common in the USA and Japan while genotypes 2, 5 and 6 have only been found in South America and genotypes 4 and 7 are common in Russia and Eastern Europe [36]. Genotype 1 has also been described in Costa Rica in Central America [17], along with genotype 5 [36]. Of the 5 nucleotide substitutions we identified in the Caribbean strains, two were silent substitutions (G87C and T534C), two were conservative substitutions (A161G and T896C) and one was a non-conservative substitution (C1505T). The conservative substitution at residue 299 was located in A epitope and the non-conservative substitution at residue 502 was located in CTM [16].

In conclusion, we have shown for the first time that BLV occurs in the Caribbean and that at least genotype 1 is present. Further studies are indicated to determine the epidemiology and extent of infections in the islands before policies can be developed to deal with the virus in the region.

## Supporting Information

S1 Table The similarity of the BLV strains we isolated and the 81 reference sequences in GenBank. (DOCX)
