# Supplementary material for: First Molecular Characterization of Bovine Leukemia Virus Infections in the Caribbean
Source: PLoS One. 2016 Dec 15;11(12):e0168379. doi: 10.1371/journal.pone.0168379 (PMC5158060; doi:10.1371/journal.pone.0168379)
Supplement: S1 Table — (DOCX) [file pone.0168379.s001.docx]

Table S1. The similarity of the BLV strains we isolated and the 81 reference sequences in GenBank.

|  | **GenBank accession number** | **KX674367/KX674368(St. Kitts)** | **KX674369/KX674370(St. Kitts)** | **KX674371 (St. Kitts)** | **KX674372/KX674373 (Dominica)** |
| --- | --- | --- | --- | --- | --- |
| **G1** | **HE967302 (Uruguay)** | 99.3% (99.0%) | 99.3% (99.2%) | 99.4% (99.4%) | 99.4% (99.2%) |
|  | **HE967303 (Uruguay)** | 99.3% (99.0%) | 99.3% (99.2%) | 99.4% (99.4%) | 99.4% (99.2%) |
|  | **LC080653 (Paraguay)** | 99.3% (99.2%) | 99.3% (99.4%) | 99.4% (99.6%) | 99.4% (99.4%) |
|  | **LC080652 (Paraguay)** | 99.3% (99.2%) | 99.4% (99.4%) | 99.5% (99.6%) | 99.5% (99.4%) |
|  | **LC080651 (Paraguay)** | 99.3% (99.2%) | 99.4% (99.4%) | 99.5% (99.6%) | 99.5% (99.4%) |
|  | **HE967301 (Uruguay)** | 99.3% (99.4%) | 99.4% (99.6%) | 99.5% (99.8%) | 99.5% (99.6%) |
|  | **AY151262 (Brazil)** | 99.2% (99.2%) | 99.2% (99.4%) | 99.3% (99.6%) | 99.3% (99.4%) |
|  | **EF065642 (USA)** | 99.4% (99.6%) | 99.5% (99.8%) | 99.5% (100.0%) | 99.5% (99.8%) |
|  | **EF065658 (Japan)** | 99.5% (99.2%) | 99.5% (99.4%) | 99.6% (99.6%) | 99.6% (99.4%) |
|  | **EF065659 (Japan)** | 99.5% (99.2%) | 99.5% (99.4%) | 99.6% (99.6%) | 99.6% (99.4%) |
|  | **EF065656 (USA)** | 99.4% (99.2%) | 99.5% (99.4%) | 99.5% (99.6%) | 99.5% (99.4%) |
|  | **EF065641 (USA)** | 99.5% (99.6%) | 99.6% (99.8%) | 99.7% (100.0%) | 99.7% (99.8%) |
|  | **EF065644 (USA)** | 99.5% (99.4%) | 99.6% (99.6%) | 99.7% (99.8%) | 99.7% (99.6%) |
|  | **M35242 (USA)** | 99.5% (99.4%) | 99.6% (99.6%) | 99.7% (99.8%) | 99.7% (99.6%) |
|  | **M35239 (USA)** | 99.4% (99.4%) | 99.5% (99.6%) | 99.5% (99.8%) | 99.5% (99.6%) |
|  | **K02120 (Japan)** | 99.2% (99.0%) | 99.3% (99.2%) | 99.4% (99.4%) | 99.4% (99.2%) |
|  | **EF065660 (Japan)** | 99.5% (99.6%) | 99.5% (99.8%) | 99.6% (100.0%) | 99.6% (99.8%) |
|  | **EF065662 (Japan)** | 99.3% (99.4%) | 99.4% (99.6%) | 99.5% (99.8%) | 99.5% (99.6%) |
|  | **EF065640 (Costa Rica)** | 99.7% (99.6%) | 99.7% (99.8%) | 99.8% (100.0%) | 99.8% (99.8%) |
|  | **AB934282 (Japan)** | 99.4% (99.0%) | 99.5% (99.2%) | 99.5% (99.4%) | 99.5% (99.2%) |
|  | **AF547184 (Brazil)** | 99.5% (99.4%) | 99.5% (99.6%) | 99.6% (99.8%) | 99.6% (99.6%) |
|  | **EF065657 (Japan)** | 99.4% (99.6%) | 99.5% (99.8%) | 99.5% (100.0%) | 99.5% (99.8%) |
|  | **EF065651 (Japan)** | 99.5% (99.6%) | 99.5% (99.8%) | 99.6% (100.0%) | 99.6% (99.8%) |
|  | **EF065653 (Japan)** | 99.5% (99.2%) | 99.5% (99.4%) | 99.6% (99.6%) | 99.6% (99.4%) |
|  | **LC005615 (Japan)** | 99.4% (99.2%) | 99.5% (99.4%) | 99.5% (99.6%) | 99.5% (99.4%) |
|  | **LC005616 (Japan)** | 99.4% (99.2%) | 99.5% (99.4%) | 99.5% (99.6%) | 99.5% (99.4%) |
|  | **EF065646 (Japan)** | 99.5% (99.2%) | 99.6% (99.4%) | 99.7% (99.6%) | 99.7% (99.4%) |
|  | **EF065652 (Japan)** | 99.5% (99.2%) | 99.6% (99.4%) | 99.7% (99.6%) | 99.7% (99.4%) |
|  | **AF399703 (Brazil)** | 99.0% (98.4%) | 99.0% (98.6%) | 99.1% (98.8%) | 99.1% (98.6%) |
|  | **D00647 (Australia)** | 97.7% (98.0%) | 97.8% (98.2%) | 97.8% (98.4%) | 97.8% (98.2%) |
| **G2** | **AF257515 (Argentina)** | 96.0% (94.8%) | 96.1% (95.0%) | 96.1% (95.2%) | 96.1% (95.0%) |
|  | **FJ914764 (Argentina)** | 96.8% (96.6%) | 96.9% (96.8%) | 97.0% (97.0%) | 97.0% (96.8%) |
|  | **AF399704 (Brazil)** | 97.1% (97.6%) | 97.2% (97.8%) | 97.2% (98.0%) | 97.2% (97.8%) |
|  | **LC080655 (Paraguay)** | 97.3% (97.8%) | 97.4% (98.0%) | 97.4% (98.2%) | 97.4% (98.0%) |
|  | **LC080654 (Peru)** | 97.0% (97.2%) | 97.1% (97.4%) | 97.2% (97.6%) | 97.1% (97.4%) |
| **G3** | **EF065647 (USA)** | 97.1% (97.6%) | 97.2% (97.8%) | 97.3% (98.0%) | 97.3% (97.8%) |
|  | **EF065649 (USA)** | 97.1% (97.4%) | 97.2% (97.6%) | 97.3% (97.8%) | 97.3% (97.6%) |
|  | **EF065648 (USA)** | 97.3% (97.6%) | 97.4% (97.8%) | 97.4% (98.0%) | 97.4% (97.8%) |
|  | **EF065650 (Japan)** | 97.1% (97.6%) | 97.1% (97.8%) | 97.2% (98.0%) | 97.2% (97.8%) |
| **G4** | **AF503581 (Belgium)** | 96.2% (97.0%) | 96.3% (97.2%) | 96.3% (97.4%) | 96.3% (97.2%) |
|  | **M35240 (Belgium)** | 96.2% (97.0%) | 96.2% (97.2%) | 96.3% (97.4%) | 96.3% (97.2%) |
|  | **EF065638 (Belgium)** | 96.5% (97.0%) | 96.6% (97.2%) | 96.7% (97.4%) | 96.7% (97.2%) |
|  | **K02251 (Belgium)** | 96.6% (97.2%) | 96.7% (97.4%) | 96.7% (97.6%) | 96.7% (97.4%) |
|  | **JN695878 (Russia)** | 96.3% (97.2%) | 96.3% (97.4%) | 96.4% (97.6%) | 96.4% (97.4%) |
|  | **M35238 (France)** | 96.7% (97.2%) | 96.7% (97.4%) | 96.8% (97.6%) | 96.7% (97.4%) |
| **G5** | **EF065654 (Costa Rica)** | 95.7% (96.4%) | 95.8% (96.6%) | 95.8% (96.8%) | 96.0% (96.6%) |
|  | **EF065655 (Costa Rica)** | 96.0% (96.4%) | 96.0% (96.6%) | 96.1% (96.8%) | 96.1% (96.6%) |
|  | **EF065643 (Costa Rica)** | 95.6% (96.2%) | 95.6% (96.4%) | 95.7% (96.6%) | 95.7% (96.4%) |
|  | **EF065635 (Costa Rica)** | 95.9% (96.2%) | 96.0% (96.4%) | 96.0% (96.6%) | 96.0% (96.4%) |
|  | **EF065636 (Costa Rica)** | 95.9% (96.2%) | 96.0% (96.4%) | 96.0% (96.6%) | 96.0% (96.4%) |
|  | **EF065639 (Costa Rica)** | 96.9% (96.6%) | 96.9% (96.8%) | 97.0% (97.0%) | 97.0% (96.8%) |
|  | **EF065645 (Costa Rica)** | 97.0% (97.0%) | 97.1% (97.2%) | 97.2% (97.4%) | 97.1% (97.2%) |
| **G6** | **AY185360 (Brazil)** | 95.8% (96.2%) | 95.8% (96.4%) | 95.9% (96.6%) | 95.9% (96.4%) |
|  | **LC080656 (Paraguay)** | 95.9% (96.8%) | 96.0% (97.0%) | 96.0% (97.2%) | 96.0% (97.0%) |
|  | **LC080657 (Paraguay)** | 95.5% (96.8%) | 95.6% (97.0%) | 95.6% (97.2%) | 95.6% (97.0%) |
|  | **LC080658 (Paraguay)** | 95.3% (96.4%) | 95.4% (96.6%) | 95.5% (96.8%) | 95.5% (96.6%) |
| **G7** | **JN695880 (Russia)** | 96.5% (97.2%) | 96.5% (97.4%) | 96.6% (97.6%) | 96.6% (97.4%) |
|  | **JN695881 (Russia)** | 96.2% (96.8%) | 96.3% (97.0%) | 96.3% (97.2%) | 96.3% (97.0%) |
|  | **JN695879 (Russia)** | 96.5% (97.2%) | 96.5% (97.4%) | 96.6% (97.6%) | 96.6% (97.4%) |
|  | **KF801457 (Moldova)** | 96.5% (97.4%) | 96.5% (97.6%) | 96.6% (97.8%) | 96.6% (97.6%) |
|  | **KF801458 (Moldova)** | 96.5% (97.4%) | 96.5% (97.6%) | 96.6% (97.8%) | 96.6% (97.6%) |
| **G9** | **LC080659 (Bolivia)** | 97.2% (97.4%) | 97.2% (97.6%) | 97.3% (97.8%) | 97.1% (97.6%) |
|  | **LC080663 (Bolivia)** | 97.2% (97.6%) | 97.2% (97.8%) | 97.3% (98.0%) | 97.1% (97.8%) |
|  | **LC080661 (Bolivia)** | 97.2% (97.6%) | 97.3% (97.8%) | 97.4% (98.0%) | 97.2% (97.8%) |
|  | **LC080662 (Bolivia)** | 97.2% (97.6%) | 97.2% (97.8%) | 97.3% (98.0%) | 97.1% (97.8%) |
|  | **LC080660 (Bolivia)** | 97.3% (97.6%) | 97.4% (97.8%) | 97.4% (98.0%) | 97.3% (97.8%) |
|  | **LC080666 (Bolivia)** | 97.0% (97.6%) | 97.1% (97.8%) | 97.2% (98.0%) | 97.0% (97.8%) |
|  | **LC080668 (Bolivia)** | 97.0% (97.6%) | 97.1% (97.8%) | 97.2% (98.0%) | 97.0% (97.8%) |
|  | **LC080665 (Bolivia)** | 97.2% (97.6%) | 97.2% (97.8%) | 97.3% (98.0%) | 97.2% (97.8%) |
|  | **LC080667 (Bolivia)** | 97.2% (97.6%) | 97.2% (97.8%) | 97.3% (98.0%) | 97.2% (97.8%) |
|  | **LC080669 (Bolivia)** | 97.2% (97.6%) | 97.2% (97.8%) | 97.3% (98.0%) | 97.2% (97.8%) |
|  | **LC080671 (Bolivia)** | 97.2% (97.6%) | 97.2% (97.8%) | 97.3% (98.0%) | 97.2% (97.8%) |
|  | **LC080672 (Bolivia)** | 97.2% (97.6%) | 97.2% (97.8%) | 97.3% (98.0%) | 97.2% (97.8%) |
|  | **LC080673 (Bolivia)** | 97.2% (97.6%) | 97.2% (97.8%) | 97.3% (98.0%) | 97.2% (97.8%) |
|  | **LC080674 (Bolivia)** | 97.2% (97.6%) | 97.2% (97.8%) | 97.3% (98.0%) | 97.2% (97.8%) |
|  | **LC080675 (Bolivia)** | 97.2% (97.6%) | 97.2% (97.8%) | 97.3% (98.0%) | 97.2% (97.8%) |
|  | **LC080664 (Bolivia)** | 97.1% (97.6%) | 97.2% (97.8%) | 97.2% (98.0%) | 97.1% (97.8%) |
|  | **LC080670 (Bolivia)** | 97.1% (97.6%) | 97.2% (97.8%) | 97.2% (98.0%) | 97.1% (97.8%) |
